# Study of half-metallic ferromagnetism and transport characteristics of double perovskites Sr_2_AIrO_6_ (A = Y, Lu, Sc) for spintronic applications

**DOI:** 10.1039/d4ra03417a

**Published:** 2024-06-04

**Authors:** Maiza Zanib, M. Waqas Iqbal, Hamid Ullah, Badriah S. Almutairi, A. Laref

**Affiliations:** a Department of Physics, Riphah International University Lahore Campus 56000 Pakistan waqas.iqbal@riphah.edu.pk; b Department of Physics, College of Science, Princess Nourah bint Abdulrahman University P.O. Box 84428 Riyadh 11671 Saudi Arabia; c Department of Physics and Astronomy, College of Science, King Saud University Riyadh 11451 Kingdom of Saudi Arabia

## Abstract

The precise manipulation of electromagnetic and thermoelectric characteristics in the miniaturization of electronic devices offers a promising foundation for practical applications in quantum computing. Double perovskites characterized by stability, non-toxicity, and spin polarization, have emerged as appealing candidates for spintronic applications. This study explores the theoretical elucidation of the influence of iridium's 5d electrons on the magnetic characteristics of Sr_2_AIrO_6_ (A = Y, Lu, Sc) with WIEN2k code. The determined formation energies confirm the thermodynamic stability while an analysis of band structure and the density of states (DOS) reveals a half-metallic ferromagnetic character. This characteristic is comprehensible through the analysis of exchange constants and exchange energies. The current analysis suggests that crystal field effects, a fundamental hybridization process and exchange energies contribute to the emergence of ferromagnetism due to electron–spin interactions. Finally, assessments of electrical and thermal conductivities, Seebeck coefficient, power factor, figure of merit and magnetic susceptibility are conducted to assess the potential of the investigated materials for the applications in thermoelectric devices.

## Abstract 2

The precise manipulation of electromagnetic and thermoelectric characteristics in the miniaturization of electronic devices offers a promising foundation for practical applications in quantum computing.

## Graphical abstract

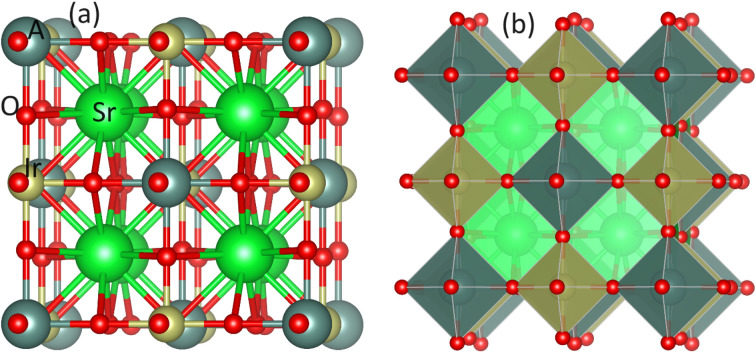

## 1. Introduction

Organic–inorganic halide double perovskites have become a promising category of materials with applications spanning diverse fields, including spintronics,^1^ ferroelectrics,^2^ optoelectronics like light-emitting diodes (LEDs)^3,4^ and photovoltaic devices,^5^ X-ray detectors,^6^ photo-detectors,^7^ and sensors.^8^ Lead-free halide double perovskites (DPs) generally expressed as A_2_BB′X_6_, comprising a combination of one monovalent and one trivalent ion, have emerged widely as environmentally friendly and stable alternatives to hazardous lead-based halide perovskites. The impressive opto-electronic characteristics of these materials can be attributed to their compositional flexibility, exciton binding energies, and dielectric properties that span several orders of magnitude.^9–11^ Among the experimentally examined materials, such as Cs_2_AgInX_6_,^12,13^ Cs_2_AgSbX_6_,^14,15^ Cs_2_InB^3+^X_6_ (B^3+^ = Sb, Bi),^16,17^ and Cs_2_AgBiX_6_ (X = Cl, Br, I),^18,19^ there has been significant interest due to their captivating properties. Among materials in this category, Cs_2_AgBiBr_6_ (ref. 20) stands out as one of the most extensively studied, exhibiting enhanced thermodynamic stability despite possessing an indirect band gap of approximately 2 eV and a power conversion efficiency around 3%.

Nevertheless, an unexplored aspect with significant potential is the manipulation of photovoltaic (PV) characteristics through the magnetic spin degrees of freedom inherent in magnetic perovskites, offering the possibility of remarkable spin-related characteristics. In this context, cubic Cs_2_AgFeCl_6_ (ref. 21) has been experimentally noted for its promising optoelectronic features and PV performance. However, the relationship between magnetic degrees of freedom and optical properties remains an avenue yet to be fully explored. The interaction between two spin degrees of freedom in these magnetic systems provides an extensive framework for adjusting the absorption range, aiming to capture the entire solar radiation spectrum. Double perovskite oxides are gaining recognition as promising candidates for the spintronic engineering due to the ability to transform traditional charge-based electronics.^22^ Spintronics leverages the intrinsic spin characteristic of electrons to transport, store and process the data, offering advantages such as significant data storage density and low power consumption.^23^ Half-metallic (HM) materials, demonstrating complete spin polarizability for electrons and holes, constitute a substantial category of materials in the field of spintronics.^24^ The accessibility of active HM compounds is essential for advancing eminent – functioning of spintronic.^25^ Oxide-based double perovskites exhibit distinctive electrical and magnetic characteristics, rendering them well-suited for spintronic applications.

With a general formula of A_2_BB′O_6_, double perovskite oxides exhibit promises as materials for spintronic applications,^26^ serving various purposes such as spin valves, magnetic storage and magnetic tunnel junctions. Mir and Gupta commenced optimization of the crystal structure and evaluated that the value of enthalpy of formation lies within the stable range, and also explored several other physical parameters. In the study of electronic band structures, Ba_2_FeNiO_6_ displayed a half-metallic (HM) response with 100% spin-polarization at the Fermi level, while Ba_2_CoNiO_6_ presented ferromagnetic semiconductor nature. The paper highlighted the influence of these materials' band structures on their thermoelectric capabilities.^27^ In their exploration of the half-metallic character in double perovskite materials Bi_2_BB′O_6_ (B, B′ = 3d transition metal). Lin *et al.* identified three prospective series of half-metals through first-principles calculations, considering generalized gradient approximation (GGA) and strong correlation effects (GGA + U) along with comprehensive structural characteristics.^28^ Further exploration and development in the field of spintronics could potentially revolutionize information technology, giving rise to more efficient and versatile spintronic tools. The aim of this study is to gain insights into the features of Sr_2_AIrO_6_ (A = Y, Lu, Sc) materials, encompassing their magnetic, elastic, thermodynamic and electronic properties.

## 2. Methodology

To investigate half-metallic ferromagnetism and mechanical characteristics of DP Sr_2_AIrO_6_ (A = Y, Lu, Sc) oxides, Wien2K software^29^ that based on PF-LAPW + lo method have been carried out. We employed PBEsol approximation to treat the exchange–correlation potential during structural optimization to obtained ground state energies of ferromagnetic (FM) and antiferromagnetic (AFM) phases. For spin polarized electronic properties, PBEsol-GGA plus modified Becke and Johnson (mBJ) potential is applied to overcome the constraints of the PBEsol method and achieve accurate bandgap calculations. We engaged the modified Becke and Johnson (mBJ) potential.^30^ In all electron's method, mBJ potential is more efficient and give accurate bandgaps compared to other potential that commonly used in computational field.^31^ The cut-off parameter, determined by the product of the muffin-tin sphere radius (*R*_max_) and the maximum plane wave (*K*_max_) was set to 8. The potential within the interstitial region was evaluated with *G*_max_ = 18 a.u^−1^.^32^ To integrate over the Brillion zone, a 12 × 12 × 12 *k*-mesh was utilized. The total energy and charge convergence criteria were set to be below 10^−4^ Ry and 10^−3^ e, respectively, for accurate results. For the calculation of electronic transport parameters like conductivities, Seebeck co-efficient and power factor, BoltzTraP code^33^ that based on semiclassical Boltzmann relations are applied. For all parameters, we used constant relaxation time approximation that is 10^−14^ s.

## 3. Results and discussion

### 3.1 Structural properties

**Fig. 1:**
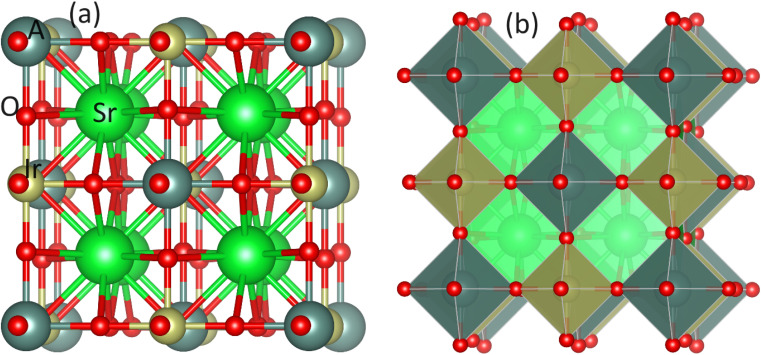
(a) Cubic unit cell of Sr_2_AIrO_6_ oxides in ball format and (b) polyhedral format using VESTA.

The Sr_2_AIrO_6_ (A = Y, Lu, Sc) double perovskite with cubic crystal structure, characterized by the space group *Fm*3*m*, can be generally expressed as A_2_BB′X_6_. The unit-cell of the double perovskite is described in Fig. 1(a) ball format and (b) polyhedral format, where the A atoms conquer 8c, B atoms conquer 4a, B′ atoms grab 4b, and X atoms are positioned at the 24e Wyckoff positions. The ground state lattice parameter *a*_0_ (Å) is theoretically computed through the CIF calculation by using the unit cell.

**Fig. 2:**
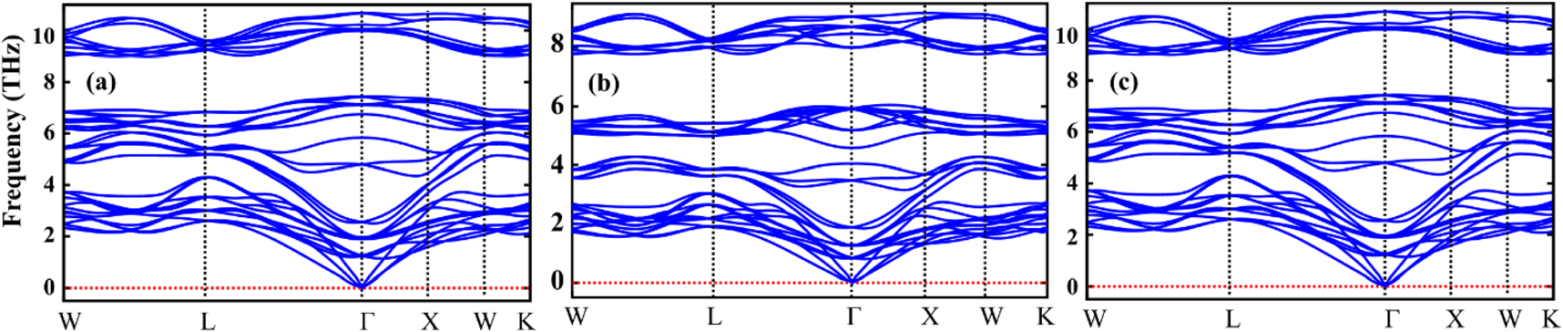
Computed phonon dispersion plot for DP oxides (a) Sr_2_YIrO_6_, (b) Sr_2_LuIrO_6_ and (c) Sr_2_ScIrO_6_.

Table 1 represents the calculated bulk moduli and ground state lattice constant. The FP-LAPW approach in DFT was used to compute the ground state energy for all materials. The Birch–Murnaghan relation was used to fit the data from the energy-volume optimization graph to obtain the lattice constant and bulk modulus, as described in ref. 34. The phonon dispersion spectra of the crystal structure of Sr_2_AIrO_6_ (A = Y, Lu, Sc) has been governed to ensure dynamical stability. The absence of imaginary frequency at the *Γ*-point has been verified through the observed phonon spectra and the examination of greater even directions (*W*–*L*–*Γ*–*X*–*W*–*K*) in the Brillouin zone, validating those materials are stable dynamically.^35,36^ All double perovskites (DPs) Sr_2_AIrO_6_ (A = Y, La, Sc) consist of 10 atoms in the primitive unit cell and are associated with 3 acoustic branches and 27 optical modes, as demonstrated in Fig. 2(a)–(c). Phonon gaps are absent among the optical phonon branches due to the marginal mass variance among Sr, A, Ir, and O atoms. Additionally, phonon simulations were conducted using the earlier optimized configurations to confirm the dynamical stability of the investigated spinels. Dynamic stability holds important significance in spintronics, providing insights into any anomalies within the examined crystal structure.

**Table 1:** Calculated parameters using PBEsol-GGA, *a*_0_ (Å): lattice constant, *B*_0_ (GPa): bulk modulus, Δ*H* (eV): formation energy and elastic parameters of Sr_2_AIrO_6_ (A = Y, Lu, Sc)

DP oxides	*a*_0_ (Å)	*B*_0_ (GPa)	Δ*H*_f_ (eV)	*C*_11_	*C*_12_	*C*_44_	*B* (GPa)	*G* (GPa)	*Y* (GPa)	*ν*	*B*/*G*
Sr_2_YIrO_6_	8.21	162.85	−2.29	276.71	100.81	59.02	159.3	69.18	181.13	0.31	2.30
Sr_2_LuIrO_6_	8.14	167.08	−1.91	299.07	99.64	64.97	166.01	77.16	200.28	0.30	2.15
Sr_2_ScIrO_6_	8.01	173.41	−1.79	278.33	121.58	61.77	173.80	67.95	180.36	0.33	2.56

Furthermore, thermodynamic stability has also been confirmed by the formation energy (Δ*H*_f_) in electron volts (eV). The negative sign (−ve) of the analyzed Δ*H*_f_ indicates that energy is released during the formation of the material, affirming the thermodynamic suitability of the analyzed materials. Sr_2_ScIrO_6_ exhibits a higher value of Δ*H*_f_ compared to Sr_2_LuIrO_6_ and Sr_2_YIrO_6_ as detailed in Table 1. This observation suggests that the stability of Sc-based composition is greater than those based on Lu and Y.^37^

**Fig. 3:**
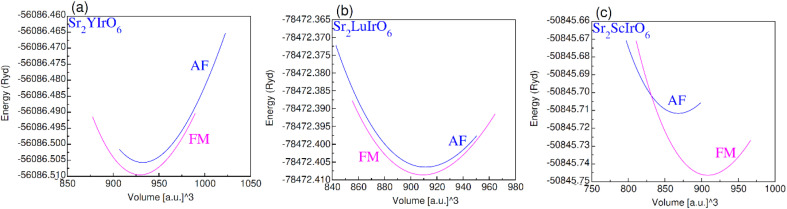
Ferromagnetic (FM) and anti-magnetic (AFM) optimization plot for (a) Sr_2_YIrO_6_, (b) Sr_2_LuIrO_6_ and (c) Sr_2_ScIrO_6_.

The plot in Fig. 3(a)–(c) illustrates the relationship between volume and total energy by using GGA. It is evident that all compositions exhibit greater stability in the ferromagnetic (FM) state compared to the anti-ferromagnetic (AF) state. This is attributed to the higher energy liberate through the formation of the ferromagnetic phase as opposed to the anti-ferromagnetic phase, emphasizing that ferromagnetic states are more stable and hold promise for practical applications.

### 3.2 Elastic properties

Calculating the mechanical properties of Sr_2_AIrO_6_ (A = Y, Lu, Sc) involves determining elastic constants that elucidate the composition's response to several stress appliances. For explaining the mechanical strength, *C*_11_, *C*_12_, and *C*_44_ elastic constants are necessary to calculate for a cubic crystal structure, and these constants are detailed in Table 1.^38^ Born stability criteria, which are essential for describing the mechanical stability of any composition, are expressed as *C*_11_ − *C*_12_ > 0, *C*_11_ + 2*C*_12_ > 0, *C*_44_ > 0 and *C*_11_ < *C*_12_. Additionally, elastic moduli including bulk (*B*), Young's (*Y*) and shear (*G*) moduli are provided in Table 1. We noted the bulk modulus values to be 159.3, 166.01, and 173.8 GPa, that comparable to computed values of bulk modulus using Birch–Murnaghan relation to be 162.85, 167.08, and 173.01 GPa for Sr_2_YIrO_6_, Sr_2_LuIrO_6_, and Sr_2_ScIrO_6_, respectively. Notably, the values of the bulk, shear, and Young's modulus for Sr_2_ScIrO_6_ are higher than those for the other two compositions. This observation indicates that Sr_2_ScIrO_6_ exhibits greater resistance to mechanical stress compared to Sr_2_YIrO_6_ and Sr_2_LuIrO_6_.^39^ Furthermore, the flexibility or brittleness of materials has been characterized using the Pugh ratio (*B*/*G*) and Poisson ratio (*ν*). The values *B*/*G* = 1.75 and *ν* = 0.26 serve as distinguishing factors between ductility and brittleness, with materials having more value than the threshold values considered ductile. The magnitude of Poisson ratio is 0.31, 0.30, and 0.33, and the values of *B*/*G* are 2.30, 2.15, and 2.56 Sr_2_YIrO_6_, Sr_2_LuIrO_6_, and Sr_2_ScIrO_6_, respectively, as outlined in Table 1. As the calculated results surpass their critical thresholds, it indicates the ductile nature of the investigated materials.^40^

A comprehensive analysis that involves crystal structure, electronic band structure, and phonon behavior alongside the mechanical properties is needed to understand the correlation between mechanical properties and thermoelectric performance. The crystal structure of Sr_2_AIrO_6_ (A = Y, Lu, Sc) double perovskites, plays an important role in determining its thermoelectric characteristics. Variations in the A-site cation (Y, Lu, Sc) can lead to different crystal structures which affect the properties like lattice parameters, bond lengths, and coordination environments, which in turn influence the transport properties. The electronic band structure directs the electrical conductivity as well as Seebeck coefficient of a material. Band engineering by altering the composition modify the band structure which affect the mobility, charge carrier concentration and density of states near the Fermi level, that are crucial for thermo-electric performance.

### 3.3 Electronic properties

**Fig. 4:**
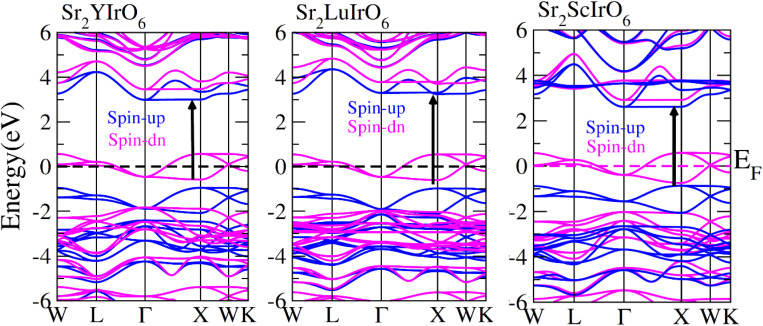
Computed band structures plot for spin up (↑) and spin down (↓) channels of Sr_2_YIrO_6_, Sr_2_LuIrO_6_ and Sr_2_ScIrO_6_ oxides.

In order to gain a deeper understanding of the electrical properties of double perovskites Sr_2_AIrO_6_ (A = Y, Lu, Sc) we generated spin-polarized band structures. The spin-polarized band structures presented in Fig. 4 offer a holistic view of the ferromagnetic characteristics of these examined compounds. In the spin-up (↑) configuration, the Fermi level is positioned between the conduction and valence bands, indicating semiconducting nature of the materials. Conversely metallic nature is observed in the spin-down (↓) configuration, because the energy states intersect the Fermi level (*E*_F_). Materials are referred as half-metallic ferromagnets that contain the electrons in one spin channel at the Fermi level, holding significant promise for uses in spintronics.

**Fig. 5:**
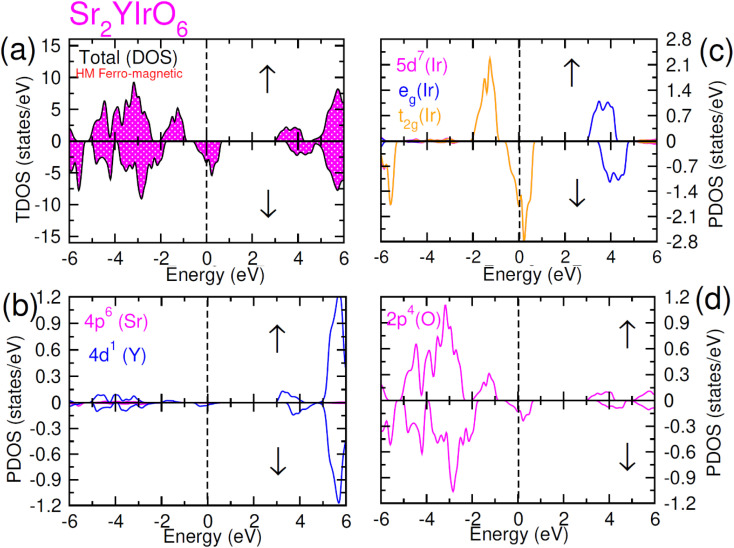
Computed density of states (DOS) plot for spin up (↑) and spin down (↓) channels (a) total DOS of Sr_2_YIrO_6_ and (b–d) their PDOS of Sr, Y, Ir and O atoms.

**Fig. 6:**
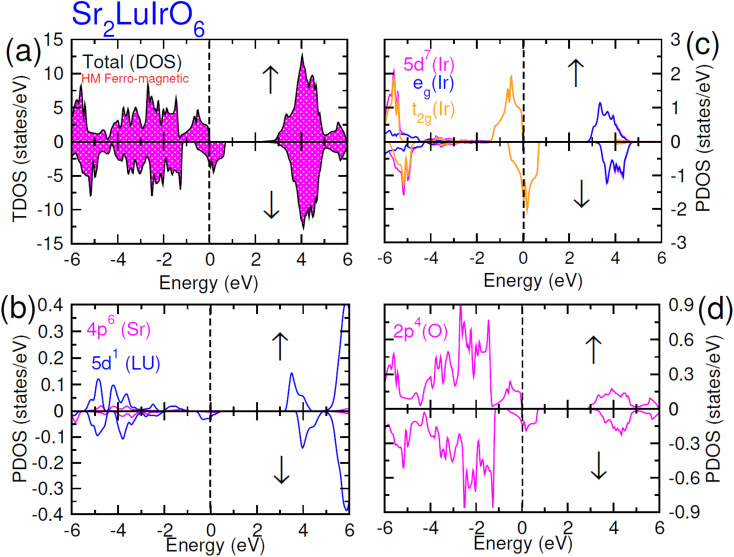
Computed density of states (DOS) plot for spin up (↑) and spin down (↓) channels (a) total DOS of Sr_2_LuIrO_6_ and (b–d) their PDOS of Sr, Lu, Ir and O atoms.

**Fig. 7:**
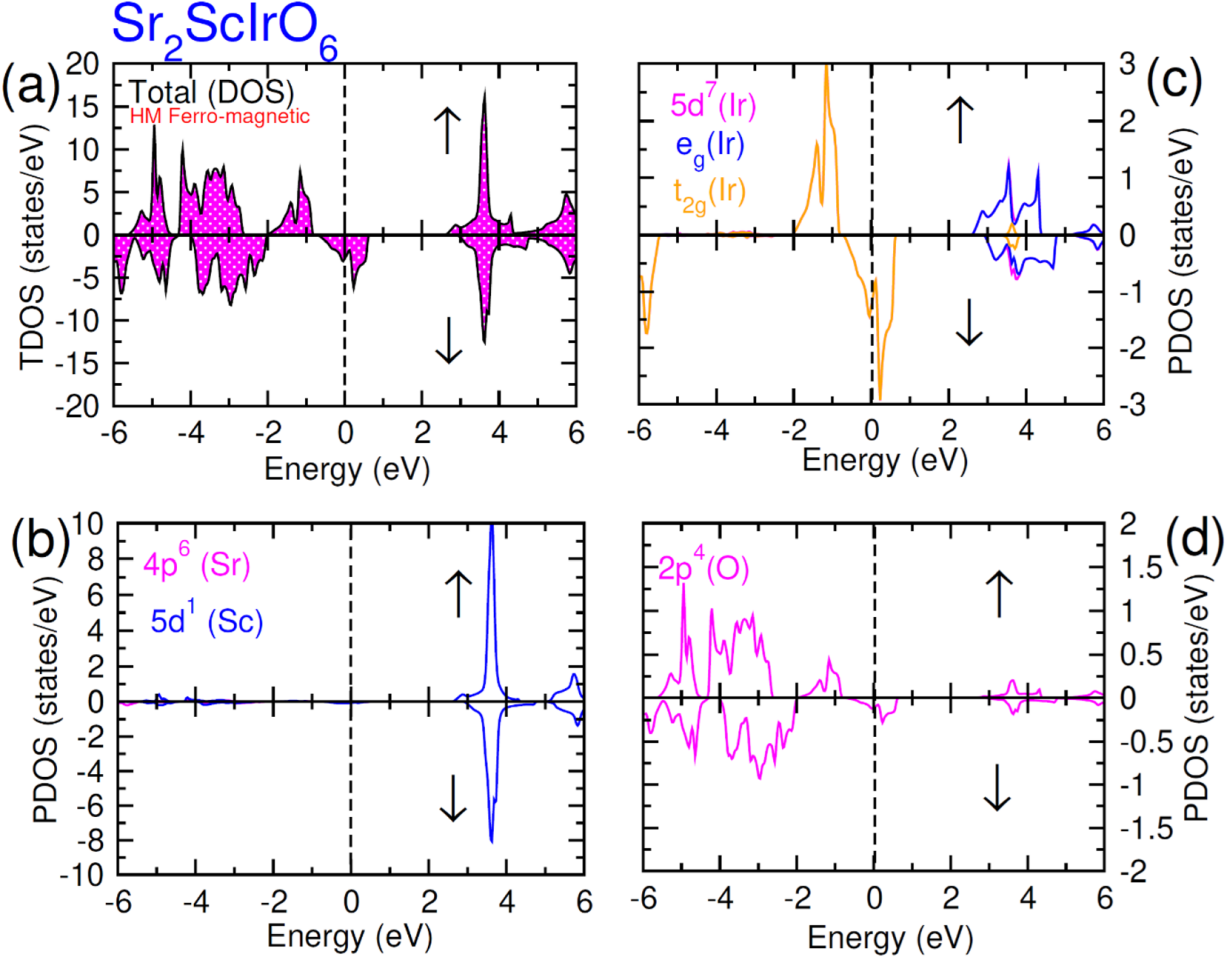
Computed density of states (DOS) plot for spin up (↑) and spin down (↓) channels (a) total DOS of Sr_2_ScIrO_6_ and (b–d) their PDOS of Sr, Sc, Ir and O atoms.

Examining the spin-polarized band structure reveals both distinctions and similarities among various configurations. A relational analysis unveils diverse characteristics, including the presence of specific energy and bands width of the half-metallic gap. For a material to be classified as a half-metallic ferromagnet, it must exhibit 100% spin polarization, a quantity calculated as follows:^41^
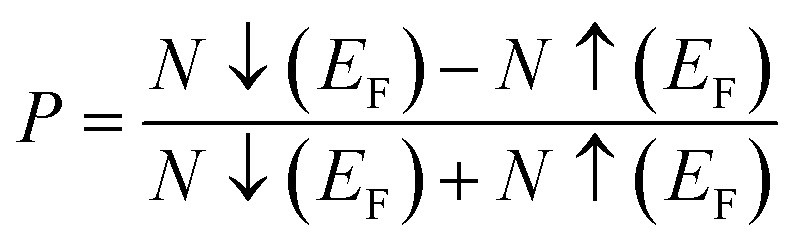
, where *N*↓(*E*_F_) and *N*↑(*E*_F_) represent the density of states for the down-spin and up-spin channels at the Fermi level, respectively. As the Fermi level resides within the bandgap in the spin-up (↑) configuration, the bandgap is determined by measuring difference of energy among the Fermi level and conduction band minima. In Sr_2_YIrO_6_, the valence band maxima are positioned at the *X* symmetry point, while the conduction band minima are located at the *X* symmetry point. This results in a separation of 3.0 eV between both band edges in the spin-up channel, indicating an indirect bandgap character. But, when Y is substituted with Lu, direct bandgap character is observed due to the shifting of both band edges along same symmetry point (*X*) having separation of 3.1 eV. When Sc replaces Lu in the composition, the band edges align at the same symmetry point, accompanied by a minor shift of the CB minima near the Fermi level, resulting in a bandgap value of 2.6 eV. In the spin-down channel, the examined materials exhibit metallic behavior. To gain a deeper understanding of the ferromagnetic fundamentals, exchange mechanisms, hybridization, total density of states (TDOS), and partial density of states (PDOS) are illustrated in Fig. 5–7(a)–(d) for Sr_2_YIrO_6,_ Sr_2_LuIrO_6_ and Sr_2_ScIrO_6_, respectively. The density of states (DOS) provides insights into the distribution of electronic states within the material across the energy scale.

In the case of Sr_2_YIrO_6_, it is noteworthy that the valence band's edge in the spin-up channel is primarily formed by the 5d-t_2g_ state of Ir, while the 4d states of Y and 2p states of O are situated deeper within the valence band. Additionally, the conduction band's edge is composed of the 5d-e_g_ state of Ir and 4d states of Y. But, in the down spin (↓) configuration, the presence of 5d-t_2g_ states of Ir and 4d states of Y around the Fermi level imparts a conducting nature. Upon replacing Y with Lu, Sr_2_YIrO_6_ exhibits a similar influence from the 5d orbital of Ir and the 2p orbital of O. The alteration in the electronic structure of Sr_2_YIrO_6_, in comparison to Sr_2_LuIrO_6_ (depicted in Fig. 6), is evident through noticeable differences in peak position and intensity.

In this scenario, there is a substantial increase in the influence from oxygen 2p-states, with these states touching the Fermi-level in the up spin (↑) channel and crossing it in the spin-down channel. Distinctive density of states (DOS) plots is observed for Sr_2_ScIrO_6_ (see Fig. 7) in contrast to Sr_2_LuIrO_6_ and Sr_2_YIrO_6_. In the up spin (↑) configuration, the edge of VB is formed by the 5d-t_2g_ state of Ir and the 2p state of O, while the edge of the CB is created by the 5d-e_g_ states of Ir and the 3d state of Sc. Conversely, in the down spin (↓) configuration, there is hybridization between the 5d-e_g_ states of Ir and the 2p states of O, defining the VB edge. These states actively contribute to characterizing the metallic nature of this composition since, in the down spin (↓) configuration, they extend beyond the Fermi level.^42^

The mingling of various states at the edges of bands is answerable for the exchange means among the electron spins. Understanding this exchange mechanism involves the calculation and estimation of exchange energies Δ(pd) and Δ(d) as well as crystal field energy (Δ*E*_cry_). The direct exchange energy, Δ(d), is determined by calculating the difference in energy between the top location of the d-state in up (↑) spin and down (↓) spin channels. Conversely, the indirect exchange energy, Δ(pd), is assessed by calculating the difference in electron energy existence in p and d orbits within the identical down spin (↓) configuration. Another approach to determine Δ(pd) involves observing the position of the p state of Y/Lu/Sc in the down spin (↓) configuration (see Fig. 5–7). This provides a straightforward and direct means of calculating exchange interaction energy.^43^ In the case of iridium, the t_2g_ state, referred to as the bonding state, experiences a force from the crystal field, leading it to lower energies. On the other hand, the e_g_ (Ir) state, known as the anti-bonding state, is impelled to higher energy levels due to the crystal field. Hence, the difference of energy between the t_2g_ and e_g_ states is the resultant of Δ*E*_cry_. For a main ferromagnetic character, the value of Δ*E*_cry_ should be less than that of Δ(d). The disparity between Δ(d) and Δ*E*_cry_ plays a vital role in governing the exchange interaction, contributing to the modification of the magnetic characteristics in various compositions.^44^ Another key parameter is the exchange energy Δ(pd), initiated by the interaction of the 5d-state of Ir and the 3d/4d/5d state of Y/Lu/Sc. The (−ve) magnitude of Δ(pd) confirms that the down spin (↓) channel is responsible for inducing ferromagnetism. Examining the electronic configurations of Ir (4f^14^5d^7^6s^2^) reveals that the 4f-state is occupied and rendering it non-contributory to the magnetic retort.^45^ Consequently, the ripping of states at band edges, specifically the CB minimum (Δ*E*_C_) and VB maximum (Δ*E*_V_), can be identified by the *N*_o_*α*, *N*_o_*β* exchange constants. These constants calculate s–d and p–d exchanges and is expressed as follows:^46^
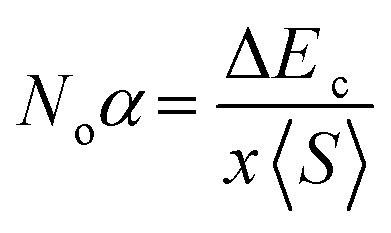
; 
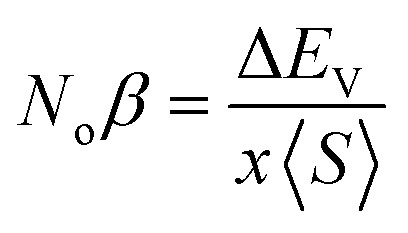
, in this context, Δ*E*_c_ = *E*_c_↓ − *E*_c_↑, signifies the energy difference at the conduction minima between the spin-up (↑) and spin-down (↓) configurations. Similarly, Δ*E*_V_ = *E*_V_↓ − *E*_V_↑, represents the energy disparity at the valence maxima in the spin-up (↑) and spin-down (↓) channel. Here, *x* denotes the contents, and *S* stands for the average value of the magnetic moment. The negative magnitude of *N*_o_*β* indicates that the spin-down (↓) configuration is conducive to ferromagnetism. Table 2 represents all the calculated exchange energy parameters.^47^

**Table 2:** Calculated crystal field energy (Δ*E*_crystal_), direct exchange Δ_*x*_(d), indirect exchange Δ_*x*_(d) and the exchange constants (*N*_o_*α* and *N*_o_*β*) of DPs Sr_2_AIrO_6_

	(Δ*E*_crystal_)	Δ_*x*_(d)	Δ_*x*_(pd)	*N*_o_ *α*	*N*_o_ *β*
Sr_2_YIrO_6_	2.8	5.2	−0.9	−0.42	−0.75
Sr_2_LuIrO_6_	2.6	4.3	−0.8	−0.09	−0.71
Sr_2_ScIrO_6_	2.4	4.0	−0.6	−0.18	−0.54

Table 3 presents the computed values of magnetic moments for individual atoms and their resulting net magnetic moments. The increased Curie temperature and the presence of vacancies at X/Ir-sites play a role in influencing the magnitude of magnetic moments. The sustained ferromagnetism over the long term is attributed to the interplay between unpaired electrons within the material and the local magnetic moment, aligning with the RKKY exchange interaction model.^48^ This contact serves as a crucial indicator frequently cited in the literature for comprehending the magnetic nature of the compound. The spin–orbit coupling in Z_2_TaCl_6_ (Z = Cs, Rb, and K) is explored by Ishikawa *et al.* and observed that the intensity of this coupling is contingent upon (i) significant ionicity, (ii) Jeff = 3/2 of 5d electrons, and (iii) the octahedral environment of halide ions.^49^ The impact of 5d-electrons on magnetoresistance and ferromagnetism in Sr_2_Fe(Mo/W/Re)O_6_ has been investigated by Fang *et al.*, revealing that the magnetic moment values are 0.39*μ*B for Mo, 0.22*μ*B for W, and −0.86*μ*B for Re.^50^ In comparison larger unquenched magnetic moments has been observed for 3d and 5d electrons, fostering solid coupling and enhanced magnetic nature.^51^ The net magnetic moments with integral values confirm 100% spin polarization, with the observed values detailed in Table 3.

**Table 3:** The total and the local magnetic moments (*μ*_B_) calculated for DPs Sr_2_AIrO_6_

	Total (*μ*_B_)	Int. (*μ*_B_)	Sr (*μ*_B_)	A (*μ*_B_)	Ir (*μ*_B_)	O (*μ*_B_)
Sr_2_YIrO_6_	1.9999	0.0456	−0.0051	−0.018	1.1986	0.131
Sr_2_LuIrO_6_	2.0000	0.0672	−0.0001	−0.042	1.1179	0.144
Sr_2_ScIrO_6_	1.9999	0.0680	−0.0007	−0.044	1.1184	0.146

### 3.4 Transport properties

**Fig. 8:**
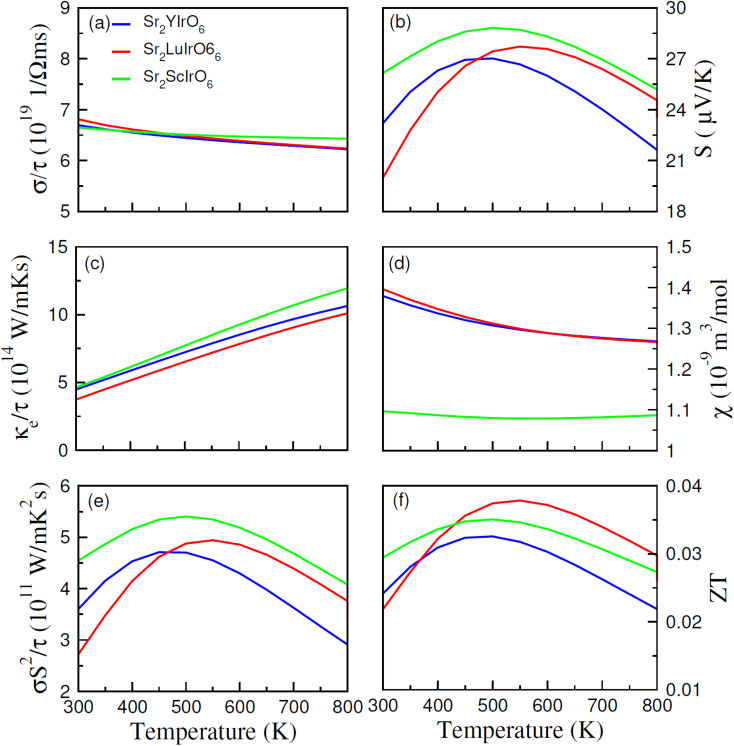
Calculated thermoelectric parameters such as (a) electrical, (b) Seebeck coefficients (c) thermal conductivities, (d) magnetic susceptibility (*χ*), (e) power factor (PF) and (f) figure of merit (*ZT*) against temperature (300–800 K) for DPs Sr_2_AIrO_6_.

The thermoelectric properties of Sr_2_AIrO_6_ (A = Y, Lu, Sc) have been examined within the temperature range of 200–800 K. The analysis of thermoelectric characteristics involves the calculation of figure of merit (*ZT*), power factor (*σS*^2^/*τ*), magnetic susceptibility (*χ*), Seebeck coefficient (*S*), electrical conductivity (*σ*/*τ*) and thermal conductivity (*κ*_e_/*τ*). A desirable thermoelectric material should exhibit a higher extent of Seebeck coefficient (*S*) and electrical conductivity (*σ*/*τ*) along with a lower level of thermal conductivity (*κ*_e_/*τ*). The BoltzTraP code was used to examine the thermoelectric features of Sr_2_AIrO_6_ (A = Y, Lu, Sc), and several factors are illustrated in Fig. 8(a)–(f).

The BoltzTraP code is employed to investigate the thermoelectric characteristics of Sr_2_AIrO_6_ (A = Y, Lu, Sc). In these calculations, the average time between consecutive collisions is referred as relaxation time, with value 10^−14^ s. The provided relationships are utilized to analyze the Seebeck coefficient, thermal conductivity (*κ*_e_/*τ*), and net electrical conductivity (*σ*/*τ*), considering their values for up (↑) and down (↓) spin channel.^52,53^ The electrical conductivity describes the potential difference response for charge carriers. The electrical conductivity values for the examined materials fell within the range of 10^19^ (Ω ms)^−1^ and showed constant decrease as temperature rises (see Fig. 8(a)). Specifically, at 200 K, the values were 7.5 × 10^19^ (Ω ms)^−1^, 7.0 × 10^19^ (Ω ms)^−1^ and 8.5 × 10^19^ (Ω ms)^−1^ for Sr_2_YIrO_6_, Sr_2_LuIrO_6_, and Sr_2_ScIrO_6_, Subsequently, at a higher temperature of 300 K, the values decreased to 6.5 × 10^19^ (Ω ms)^−1^ for Sr_2_YIrO_6_, Sr_2_LuIrO_6_ and 6.6 × 10^19^ (Ω ms)^−1^ for Sr_2_ScIrO_6_. This temperature-dependent increase in electrical conductivity values indicates the semiconducting nature of these materials. Furthermore, a comparative analysis reveals variations in *σ* with changes in Y/Lu/Sc, with the maximum value observed for Sr_2_ScIrO_6_, potentially attributed to its the rapid mobility of charge carriers and smaller ionic core size. Thermal conductivity refers to the transfer of charge-carriers induced through thermal gradient, encompassing both lattice and electronic components, known as the lattice part and electronic part of thermal conductivity, respectively. As the BoltzTraP code does not consider the lattice part so only the electronic part is computed of thermal conductivity in the current investigation, as illustrated in Fig. 8(c). The values of (*κ*/*τ*) fall within the span of 10^15^ (W m^−1^ K^−1^ s^−1^).

It demonstrated a linear increase with rising temperature, reaching values of *κ*_e_/*τ* at 200 K such as 4.8 × 10^15^ (W m^−1^ K^−1^ s^−1^) for Sr_2_YIrO_6_, and Sr_2_LuIrO_6_ and 3.50 × 10^15^ (W m^−1^ K^−1^ s^−1^) for Sr_2_ScIrO_6_. Though, these values rose to 12 × 10^15^ (W m^−1^ K^−1^ s^−1^) for Sr_2_YIrO_6_, and Sr_2_LuIrO_6_ and 11 × 10^15^ (W m^−1^ K^−1^ s^−1^) for Sr_2_ScIrO_6_ at 800 K. It is crucial for optimal performance that *κ*_e_/*τ* remains lower than that of *σ*/*τ*.^54^ The observed ratio of (*σ*/*τ*) to (*κ*/*σ*) is approximately 10^−6^ in this study. The Seebeck coefficient (*S*) quantifies the potential difference created among the ends of a material for a unit temperature difference, and its measured values are shown in Fig. 8(b). The positive Seebeck coefficient indicate that the holes are majority of charge carriers, classifying the examined materials as p-type semiconductors. The value of Seebeck coefficient (*S*) are 23 for Sr_2_YIrO_6_, 27 for Sr_2_LuIrO_6_ and 17 μV K^−1^ for Sr_2_ScIrO_6_ at 200 K. As the temperature rises to 550 K, these values increased to 25, 28, and 26 μV K^−1^ for Sr_2_YIrO_6_, Sr_2_LuIrO_6_ and Sr_2_ScIrO_6_, respectively.^55^

Magnetic susceptibility (*χ*) measures the extent to which a material becomes magnetized when subjected to an external magnetic field, and this temperature-dependent response for Sr_2_AIrO_6_ (A = Y, Lu, Sc) is depicted in Fig. 8(d). At lower temperatures, the magnetic susceptibility (*χ*) tends to be least at lower temperatures, attributed to alignment of magnetic-moments. The limited thermal energy establishes a long-term order in magnetism, resulting in reduced magnetic susceptibility. As temperature rises, the alignment of magnetic movements is disrupted, weakening their contact and rendering them more responsive to applied magnetic fields. Consequently, there is rise in *χ* as the temperature rises. Therefore, an analysis of the magnetic susceptibility with varying temperature offers a deeper perception into the magnetic characteristics and interactions of magnetic moments of the investigated materials. This observed behavior signifies a shift from well-defined ferromagnetic states at lower temperatures to less organized paramagnetic states at higher temperatures. Sr_2_ScIrO_6_ exhibited the maximum magnitude of *χ*, due to increased exchange constants influenced by the charge carrier concentration.^56^ The power factor (PF), expressed as *S*^2^*σ*/*τ* and reflecting the thermoelectric efficiency of the material, is depicted against temperature in Fig. 8(e). The trend of the power factor aligns with the electrical conductivity, emphasizing the highly conductive nature of the material. The observed PF is 3.6 × 10^11^ (W m^−1^ K^−2^ s^−1^), 4.55 × 10^11^ (W m^−1^ K^−2^ s^−1^) for Sr_2_YIrO_6_, Sr_2_LuIrO_6_ and 2.65 × 10^11^ (W m^−1^ K^−2^ s^−1^) for Sr_2_ScIrO_6_. Its magnitude increases with rising temperature in the examined materials, reaching its peak in Sr_2_ScIrO_6_. The capacity of a material to attain electrical energy from heat is vital in assessing its suitability for thermoelectric applications, quantified through the figure of merit (*ZT*). Generally, *ZT* demonstrate intricate temperature-dependence, with electronic band structure, scattering mechanisms, and carrier concentration which govern conversion efficiency, particularly at lower temperatures.

The unique electronic and crystal structures found in various materials, such as Sr_2_ScIrO_6_ (A = Y, Lu, Sc), contribute to variations in their respective *ZT* values. Sometime, the ratio of electrical conductivity to relaxation time (*σ*/*τ*) decrease as temperature rises, as carriers interact more strongly with phonons, potentially leading the figure of merit. The Seebeck coefficient, which fluctuates with temperature because of the variations in scattering-mechanisms, carrier concentration, and mobility, also influences the *ZT* magnitude. Moreover, thermal conductivity plays a pivotal role in determining *ZT* magnitude, with materials possessing lower thermal conductivity values being highly desirable for defining the overall *ZT* magnitude. The observed increase in *ZT* with rising temperature suggests that the examined materials hold promise for different thermoelectric uses.^57^

Sr_2_AIrO_6_ (A = Y, Lu, Sc) materials can exhibit spontaneous polarization due to the presence of oxygen octahedral distortion and transition metal ions. The electronic band structure influences the electrical conductivity and the Seebeck coefficient which is due to this polarization. The magnitude and direction of this polarization can enhance the thermoelectric performance. The main reason of the crystal field effects is the existence of d-orbital of transition metal ions along with the A-site cation of the Sr_2_AIrO_6_. These splitting can influence the density of states near the Fermi level and thus the thermal transport as well as electrical characteristics. It is possible to adjust the thermoelectric characteristics through the tunning of crystal fields factors by structural modifications or doping. The ferromagnetic ordering in Sr_2_AIrO_6_ due to the presence of magnetic transition metal ions (Ir) and their interactions. Ferromagnetism introduces spin-dependent transport phenomena that potentially enhance or reduce the thermoelectric performance depending on the specific material characteristics and functionality.

## 4. Conclusion

In this recent investigation, we have comprehensively examined the crystal structure, electronic band structure, as well as the optical and thermoelectric features of Sr_2_AIrO_6_ (A = Y, Lu, Sc) double perovskites through the application of DFT, utilizing the WIEN2K code. Initially, the structure is examined in both ferromagnetic and anti-ferromagnetic phases, confirming the greater stability of the ferromagnetic phase for studied oxide double perovskites. Also, cubic structural stability of these oxides is confirmed through phonon dispersion plot. In the ferromagnetic phase, Sr_2_AIrO_6_ (A = Y, Lu, Sc) exhibited formation energies of −2.29 eV, −1.91 eV, and −1.79 eV, respectively, substantiating their thermodynamic stability. The spin-polarized density of states (DOS) confirmed a 100% spin polarization. The existence of fundamental hybridization was observed due to the magnetic-moments exhibiting greater Curie temperatures. The hybridization of exchange energies and valence electrons has confirmed that the origin of ferromagnetism lies in electronic spin rather than clustering. The main result of quantum confinement caused in negative (−ve) values for exchange energy Δ(pd), and exchange coefficients. The results of the Seebeck coefficient revealed that these materials exhibited p-type semiconducting behavior. The temperature-dependent figure of merit and power factor increased with rising temperature, revealing the potential of examined materials for thermoelectric uses.

## Author contributions

Muhammad Waqas Iqbal, Badriah S. Almutairi and Maiza Zanib wrote the manuscript. Maiza Zanib and Muhammad Waqas Iqbal, Badriah S. Almutairi, A. Laref and Hamid Ullah worked on data collection, analysis, and interpretation of results. All authors discussed the progress of research and reviewed the manuscript.

## Conflicts of interest

The authors declare that they have no known competing financial interests or personal relationships that could have appeared to influence the work reported in this paper.

## Supplementary Material
